# Diet-Driven Epigenetic Alterations in Colorectal Cancer: From DNA Methylation and microRNA Expression to Liquid Biopsy Readouts

**DOI:** 10.3390/biomedicines14020267

**Published:** 2026-01-24

**Authors:** Theodora Chindea, Alina-Teodora Nicu, Gheorghe Dănuț Cimponeriu, Bianca Galateanu, Ariana Hudita, Mirela Violeta Șerban, Remus Iulian Nica, Liliana Burlibasa

**Affiliations:** 1 Faculty of Biology, University of Bucharest, 050663 Bucharest, Romania; t.chindea20@s.bio.unibuc.ro (T.C.); bianca.galateanu@bio.unibuc.ro (B.G.); ariana.hudita@bio.unibuc.ro (A.H.); mirela.serban@drd.unibuc.ro (M.V.Ș.); liliana.burlibasa@bio.unibuc.ro (L.B.); 2 Faculty of Midwifery and Nursing, Carol Davila University of Medicine and Pharmacy, 020021 Bucharest, Romania; remus.nica@umfcd.ro; 3 “Dr. Carol Davila” Central Military Emergency University Hospital, 010825 Bucharest, Romania

**Keywords:** colorectal cancer, liquid biopsy, epigenetic biomarkers, microRNA, DNA methylation, methylated microRNAs, Western diet, Mediterranean diet, bioactive compounds

## Abstract

The escalating incidence of colorectal cancer (CRC), particularly the alarming rise in early-onset cases, necessitates a paradigm shift from a purely genetic perspective to a broader investigation of promising pathways. This review explores the “nutri-epigenetic” interface, positioning liquid biopsy as a critical technology for translating dietary impacts into actionable clinical biomarkers. We contrast the molecular consequences of the Western dietary pattern, characterized by methyl-donor deficiency and pro-inflammatory metabolites, with the protective mechanisms of the Mediterranean diet. Mechanistically, we detail how Western-style diets drive a specific “epigenetic double-hit”: promoting global DNA hypomethylation (destabilizing LINE-1) while paradoxically inducing promoter hypermethylation of critical tumour suppressors (*MLH1*, *APC*, *MGMT*) and silencing tumour-suppressive microRNAs (miR-34b/c, miR-137) via methylation of their encoding genes. Conversely, we highlight the capacity of Mediterranean bioactive compounds (e.g., resveratrol, curcumin, butyrate) to inhibit DNA methyltransferases and restore epigenetic homeostasis. Bridging molecular biology and clinical utility, we demonstrate how these diet-sensitive signatures, specifically circulating methylated DNA and dysregulated microRNAs, can be captured via liquid biopsy. We propose that these circulating analytes serve as dynamic, accessible biomarkers for monitoring the molecular progression toward a carcinogenic state, thereby establishing a novel framework for personalized risk stratification and validating the efficacy of preventive nutritional strategies.

## 1. Introduction

In the 21st century, colorectal cancer (CRC) remains one of the most prevalent and lethal malignancies worldwide. According to Bray et al. 2024, approximately 1.93 million new cases and 904,000 deaths were attributed to CRC, maintaining its status as the third most common cancer and the second leading cause of cancer-related death globally [1]. Europe bears one of the heaviest burdens, accounting for 22.4% of global cancer cases and 20.4% of deaths despite having only 9.6% of the global population [1,2]. Although the notable improvements in screening and therapeutic approaches are undeniable, incidence and mortality remain substantial, and both are projected to increase further in the coming decades, driven by population aging and demographic growth [3,4]. Acknowledging the heterogeneity of European populations is crucial, as CRC-related mortality in 2021 showed marked geographic variability, ranging from 3.4% of all deaths in Croatia and 3.3% in Spain, to as low as 1.3–1.9% in Turkey, Bulgaria, Romania, Cyprus, Latvia, and Greece [1,2,4,5,6].

Santucci et al. projected that, across the European Union, age-standardized mortality rates for colorectal cancer would decline in 2024 compared to 2018, by approximately 6.5% in men and 4.3% in women [7]. Current data largely supports this downward trajectory in older populations, though it contrasts sharply with the rising mortality observed in early-onset cases [1,7].

Traditionally and canonically considered a disease of old age, CRC has shown a worrying rise among younger individuals (i.e., under 50 years of age) in recent years, particularly since the mid-1990s. This occurrence is now termed early-onset colorectal cancer (eoCRC) [8]. Data from the United States highlights the severity of this shift: CRC has risen to become the leading cause of cancer death in men under 50 and the second leading cause in women [8]. In several high-income regions, particularly Northern Europe and North America, eoCRC incidence has been increasing by 1–3% annually, with some countries reporting even steeper rises for colon cancer (up to 6–9% per year) [3,9,10,11,12]. The causes of this shift are multifactorial, involving both genetic predisposition and dynamic environmental exposure, including unhealthy dietary patterns, obesity, sedentary behaviour, and metabolic disturbances. These factors not only promote inflammation and oxidative stress but may also cause epigenetic changes that speed up the development of cancer at younger ages [13,14,15,16].

Incidence patterns reveal a stark geographic dichotomy: regions with Westernized diets (high in ultra-processed foods and red meats) exhibit the highest rates, whereas Mediterranean populations, traditionally adherent to polyphenol- and fiber-rich diets, have maintained lower disease burdens, though this advantage is eroding as dietary habits shift [9].

This means that understanding the cause of CRC requires looking beyond traditional risk factors to the epigenetic mechanisms that control gene-environment interactions. DNA methylation, carried out by DNA methyltransferases (DNMTs) at CpG islands, represents an essential mechanism in silencing tumour suppressor genes [17]. At the same time, microRNAs (miRNAs) allow fine post-transcriptional control, processed through the canonical Drosha/Dicer pathway. Mature miRNAs direct the RNA-induced Silencing Complex (RISC) to block target messenger RNAs translation or trigger their destruction [17,18].

Notably, these two epigenetic layers are functionally intertwined. A significant proportion of miRNA genes are located within CpG island-rich regions, rendering them susceptible to the same DNA hypermethylation machinery that silences protein-coding genes. This epigenetic silencing of non-coding RNA thus represents a multilayer regulatory interface where environmental stimuli can simultaneously disrupt multiple signalling pathways [19,20,21].

In this narrative review, we will explore the complex interplay between modifiable risk factors, particularly dietary patterns, and the colorectal epigenome. We will present how specific nutrients modulate DNA methylation and microRNA expression, including the methylation of miRNA genes themselves, to provide a comprehensive overview of how lifestyle choices can effectively reprogram the molecular landscape of colorectal cancer.

## 2. Staging and Classification

The CRC staging framework is based on the TNM (T—primary tumour, N—regional lymph nodes’ status, and M—distant metastases) classification [22].

The World Health Organization’s (WHO’s) classification of colorectal carcinomas presents five categories: (1) adenocarcinoma, (2) adenosquamous carcinoma, (3) squamous cell carcinoma, (4) undifferentiated carcinoma, and (5) spindle cell carcinoma. By far, the most common type of CRC is adenocarcinoma, which accounts for about 90% of all colorectal carcinomas. In contrast, colorectal malignancies such as adenosquamous, squamous cell, or undifferentiated carcinomas have a low incidence, with spindle cell carcinomas being the rarest type [23,24,25,26].

At the molecular level, CRCs can be classified using a gene-expression-based system, the Consensus Molecular Subtypes (CMS), or by other genetic/epigenetic criteria.

The CMS stratification, often called the “gold standard,” consists of CMS1 (immune subtype, approximately 14% of cases), CMS2 (canonical subtype, 37%), CMS3 (metabolic subtype, 13%), and CMS4 (mesenchymal subtype, 23%). Heterogeneous samples with differing characteristics are classified as mixed or indeterminate samples (14%). CMS1 subtype exhibits a strong immune response and widespread hypermethylation, CMS2 subtype is associated with WNT and MYC signalling activation, CMS3 is characterized by metabolic dysregulation and KRAS-activating mutations, while CMS4 manifests an upregulation of the TGFB pathway [27,28].

Tumour genetic and epigenetic profiling highlights three main pathways: chromosomal instability (CIN), microsatellite instability (MSI), and the CpG island methylator phenotype (CIMP). CIN involves large-scale chromosomal gains, losses, and rearrangements, leading to aneuploidy and accounting for most non-hypermutated CRCs. MSI results from defects in the DNA mismatch repair (MMR) system, leading to the accumulation of mutations in microsatellite regions; tumours are further categorized as MSI-high (MSI-H) or MSI-low (MSI-L), with MSI-H tumours often showing better prognosis and response to immunotherapy. CIMP is characterized by widespread promoter hypermethylation, leading to gene silencing, and is frequently associated with MSI-H tumours and specific mutations such as in *BRAF* [29].

Crucially, contemporary practice routinely assesses driver mutations (*KRAS*, *NRAS*, *BRAF*) and EGFR status, which are pivotal for prognosis and therapeutic stratification [27,28]. Expanded *RAS* testing is now mandatory for anti-EGFR therapy eligibility, while *BRAF* V600E mutations signal aggressive disease, often overlapping with the CIMP-high/MSI-H phenotype [16,27]. Integrating these biomarkers, increasingly via liquid biopsy, is essential for refining TNM staging and guiding precision treatment [1,16].

Understanding these molecular classifications is crucial, given that different risk factors, namely dietary ones and specific nutrients, have been shown to differentially impact these pathways, suggesting that dietary intervention may be most effective against particular tumour subtypes (e.g., CIMP-high tumours).

## 3. Risk Factors

Colorectal cancer is a multifactorial disease shaped by both inherited and acquired alterations, which can be broadly classified into non-modifiable and modifiable risk factors [30,31]. Among the non-modifiable determinants, age, sex, and genetic susceptibility play central roles. The incidence of CRC increases sharply after the age of 50, and men consistently exhibit a higher risk than women [31].

Inherited syndromes, such as familial adenomatous polyposis (FAP) and Lynch syndrome (LS), make up about 5% of all cases, while a positive family history significantly raises individual risk. These conditions result from distinct germline mutations that increase the risk of cancer development via well-understood molecular pathways [30].

Familial adenomatous polyposis (FAP) is an autosomal dominant syndrome primarily driven by inherited heterozygous germline mutations in the *Adenomatous Polyposis Coli* (*APC*) tumour suppressor gene [32]. These genetic alterations often manifest as protein-truncating nonsense or frameshift mutations (e.g., p.S1281, p.E1538, or p.V677Sfs*3) that result in a premature termination of translation and loss of protein function [33]. Functional *APC* acts as a critical negative regulator of the Wnt signalling pathway, while its genetic inactivation leads to the accumulation of β-catenin and the constitutive activation of Wnt signalling, which triggers genomic instability and uncontrolled cell proliferation. This progression aligns with Knudson’s two-hit hypothesis, where the inherited germline mutation serves as the first hit, and polyp formation is initiated following the acquisition of a somatic second hit in the wild-type allele. Notably, the specific location of the germline mutation influences the type of the somatic second hit selected during tumorigenesis and directly correlates with the severity of the clinical phenotype [33,34].

Lynch syndrome (LS), also known as hereditary non-polyposis colorectal cancer (HNPCC), is an autosomal dominant condition caused by pathogenic germline variants in the DNA mismatch repair (MMR) genes *MLH1*, *MSH2*, *MSH6*, and *PMS2*, or deletions in *EPCAM* [35]. Among these, *MLH1* and *MSH2* variants are the most frequent causes of the syndrome [35,36]. A critical feature of the MMR system is the interdependence of protein stability, namely, the MLH1 protein is required to stabilize PMS2 by forming the MutLα heterodimer [36,37]. Consequently, a germline mutation in *MLH1* often results in secondary loss of PMS2 protein expression due to proteolytic degradation [37]. This genetic failure leads to the accumulation of uncorrected errors in repetitive DNA sequences, via the loss of mismatch repair capability, resulting in the MSI phenotype characteristic of LS tumours [35].

Emerging evidence also suggests that early-life exposures (e.g., antibiotic use during childhood or pregnancy, gestational diseases, and early introduction of formula feeding) may influence the intestinal microbiome and contribute to the development of early-onset CRC (eoCRC). Mechanistically, early-life dysbiosis reduces the production of key short-chain fatty acids, particularly butyrate, whose absence disrupts epithelial differentiation, promotes low-grade inflammation, and weakens epigenetic homeostasis through loss of HDAC inhibition, collectively creating a pro-tumorigenic microenvironment that predisposes to eoCRC [6,30].

**Figure 1:**
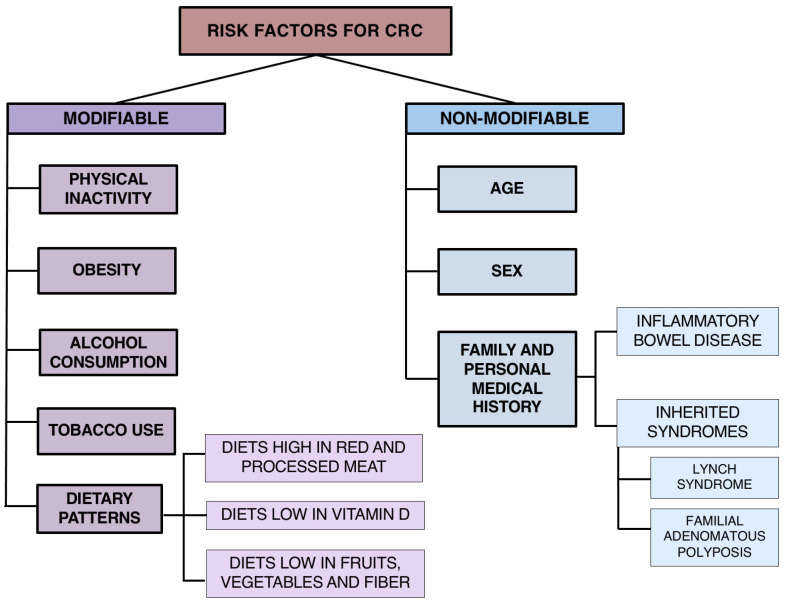
Classification of modifiable and non-modifiable risk factors associated with colorectal cancer development.

In contrast, modifiable risk factors, which can be influenced through behavioural and policy measures, are key to preventing CRC. Obesity and alcohol consumption are most strongly associated with CRC, whereas physical inactivity, smoking, high consumption of sugary drinks and processed meat, and low intake of fruits and vegetables also contribute to increased risk [30,38]. A broad classification of risk factors is shown in Figure 1.

A meta-analysis by Johnson et al. [39], involving over 66,000 CRC patients, demonstrated that the risk of CRC increases by approximately 29% for every 8 kg/m^2^ rise in BMI across the entire cohort. Crucially, the analysis revealed a highly significant sex heterogeneity: the risk for colon cancer specifically was 53% greater per 8 kg/m^2^ in men, but a statistically significant dose–response increase in risk was not found in women [39]. Obesity likely promotes tumorigenesis through insulin resistance, chronic inflammation, oxidative stress, and elevated IGF-1 levels, which stimulate cellular proliferation.

Type 2 diabetes mellitus represents a well-established but under-discussed risk factor for CRC, sharing overlapping metabolic, inflammatory, and epigenetic mechanisms with obesity. Chronic hyperglycaemia and insulin resistance promote oxidative stress, low-grade inflammation, and aberrant DNA methylation patterns. In parallel, diets rich in advanced glycation end products (AGEs), abundant in processed and high-temperature-cooked foods, activate the AGE–RAGE signalling axis, further amplifying NF-κB–driven inflammation and DNMT recruitment [40]. These mechanisms provide a direct link between diabetic metabolic states, Western dietary patterns, epigenetic dysregulation, and colorectal carcinogenesis [41]. Conversely, regular physical activity reduces CRC risk and improves survival, with a clear inverse relationship between exercise intensity and disease incidence [42].

Among these environmental exposures, diet is the most modifiable and potent lever for influencing carcinogenic risk. Consequently, dietary patterns are among the most powerful and actionable determinants of CRC risk. The Western dietary pattern (characterized by high intakes of red and processed meats, refined grains, sweets, high-fat dairy products, low fiber intake, and sugary beverages) has been consistently linked to an increased risk of CRC [43]. This association is attributed to the formation of carcinogenic compounds such as heterocyclic amines, polycyclic aromatic hydrocarbons, and N-nitroso compounds during meat processing and high-temperature cooking, as well as to diet-induced oxidative stress and inflammation.

In contrast, the Mediterranean diet, rich in fruits, vegetables, legumes, whole grains, fish, and olive oil, has been associated with reduced CRC incidence [43,44]. Its protective effects are mediated by a high content of dietary fibers, antioxidants, and anti-inflammatory compounds, which enhance gut motility, support a healthy microbiome, and reduce oxidative DNA damage. Olive oil, the primary lipid source in this dietary pattern, has been shown to induce apoptosis and downregulate pro-tumorigenic proteins such as COX-2 and Bcl-2 [43]. Interestingly, some studies have observed sex-specific differences, with a more pronounced detrimental effect of the Western diet in females and a more substantial protective effect of the Mediterranean diet in males [43,45,46].

Altogether, these observations highlight that while genetic predisposition establishes a foundation of risk, lifestyle, and particularly diet, can profoundly modulate susceptibility through molecular and metabolic pathways. Among these, epigenetic alterations such as DNA methylation and specific microRNA synthesis represent a crucial interface linking environmental exposures to colorectal carcinogenesis, as will be explored in the following sections.

## 4. Aberrant DNA Methylation in Colorectal Carcinogenesis

The epigenome, unlike the static genome, is a dynamic system highly responsive to environmental stimuli, particularly nutrition. DNA methylation, the addition of a methyl group to the 5-carbon of cytosine residues, acts as a critical regulator of gene expression. In CRC, global hypomethylation often leads to genomic instability, while promoter-specific hypermethylation, at CpG sites, silences key tumour suppressor genes (TSGs). The dichotomy between Western and Mediterranean dietary patterns is strikingly reflected in these epigenetic landscapes [47,48,49,50,51].

### 4.1. Primary Targets of DNA Methylation in CRC

Before presenting dietary impacts, it becomes essential to understand the molecular targets. Aberrant methylation in CRC frequently silences genes, such as *MLH1*, *APC*, *p16* (*CDKN2A*), and *MGMT*, which are critical for DNA repair and cell cycle control. Promoter hypermethylation of *MLH1* (a mismatch repair gene) mimics the germline defects of Lynch syndrome, leading to MSI [52,53,54]. Methylation of *APC* (a Wnt pathway gatekeeper) allows unchecked β-catenin signalling and is commonly observed in adenomas and early carcinomas [55,56]. Silencing of *p16* (*CDKN2A*) and *MGMT* disrupts cell cycle checkpoints and DNA repair, respectively [57,58]. The secreted frizzled-related proteins, SFRP1 and SFRP2, normally inhibit Wnt/β-catenin signalling, but promoter hypermethylation silences their expression, leading to pathway activation and uncontrolled proliferation. This epigenetic silencing occurs early in tumorigenesis, making *SFRP* genes promising early detection biomarkers [59]. The loss of methylation in repetitive elements such as LINE-1 promotes chromosomal instability, which contributes to global hypomethylation, a hallmark of Western diet-associated carcinogenesis [60,61].

Collectively, these gene-specific methylation events serve not only as drivers of carcinogenesis but also as biomarkers of tumour progression and response to dietary and environmental exposures [61,62,63,64,65].

To illustrate the functional consequences of promoter hypermethylation, the *MLH1* gene serves as a paradigm in colorectal carcinogenesis. *MLH1* is a critical component of the DNA Mismatch Repair (MMR) system, and its inactivation is the predominant cause of sporadic microsatellite instability (MSI) in CRC [66]. In sporadic CRC, particularly in the context of CIMP, the *MLH1* promoter region becomes heavily methylated at specific CpG sites. This epigenetic event accounts for the majority of MMR-deficient tumours in the sporadic setting, often co-occurring with the *BRAF* V600E mutation [67,68].

Mechanistically, this process involves the recruitment of DNA methyltransferases (DNMTs), which catalyse the addition of a methyl group to the cytosine ring. This methylation creates a binding site for Methyl-CpG-binding domain proteins (MBDs), which in turn recruit histone deacetylases (HDACs) and chromatin remodelling factors. The result is a transition from an open, transcriptionally active chromatin state (euchromatin) to a condensed, inactive state (heterochromatin) [69]. This steric hindrance prevents transcription factors from binding to the *MLH1* promoter, effectively silencing gene expression. Specifically, methylation accumulates in the proximal promoter regions (often denoted as regions C and D), which correlates strongly with the loss of *MLH1* transcription [68].

**Figure 2:**
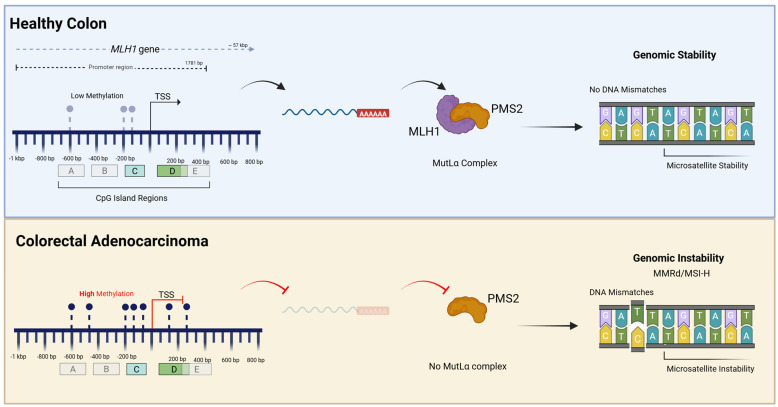
Epigenetic silencing of *MLH1* in sporadic CRC. Promoter architecture shows the *MLH1* promoter relative to the Transcription Start Site (TSS). Proximal regions C (−162 to −62 bp) and D (+88 to +260 bp) are highlighted, in blue and green, as critical zones containing regulatory elements (CCAAT/GC boxes) required for transcription factor (TF) binding. The functional mechanism is as such: Top (Healthy Colon)—The unmethylated promoter allows TF binding and active transcription (black arrow). The resulting MLH1 protein stabilizes PMS2, forming the MutLα complex that facilitates DNA mismatch repair and maintains genomic stability; Bottom (Sporadic CRC)—DNMT-mediated hypermethylation of proximal CpG sites (regions C—in blue and D—in green) induces chromatin condensation and transcriptional silencing (red blunt arrow). The subsequent loss of MLH1 causes concurrent PMS2 degradation; this deficiency in the MutLα complex prevents repair of replication errors, driving the Microsatellite Instability-High (MSI-H) phenotype. Created in BioRender.Chindea, T. (2026) https://BioRender.com/28wycew.

The clinical consequence is profound: the loss of MLH1 protein leads to a deficiency in repairing DNA replication errors, resulting in the accumulation of mutations in repetitive sequences, a phenomenon known as MSI-High. The loss of MLH1 protein expression is frequently accompanied by the concurrent loss of its heterodimer partner, PMS2 [67]. Thus, a singular epigenetic event (methylation) mimics the effect of a genetic mutation, driving a distinct carcinogenic pathway associated with proximal tumour location and specific histopathological features [67]. The complex interplay between the genomic architecture of the *MLH1* promoter and the functional consequences of its epigenetic silencing in sporadic colorectal cancer is visually summarized in Figure 2.

Furthermore, while this mechanism is classically associated with older age of onset, recent evidence suggests that constitutional methylation of the *MLH1* promoter (secondary epimutations) can also drive early-onset CRC in patients lacking germline mutations, highlighting a poorly recognized mechanism for Lynch syndrome [66].

This specific example of *MLH1* methylation can be extrapolated as a prototypical mechanism for CIMP, defined by the synchronous epigenetic silencing of multiple TSGs. The repressive cascade involving DNMTs and chromatin condensation, as described for *MLH1*, is functionally conserved across other critical loci, although the downstream consequences vary depending on the targeted pathway. For instance, *MGMT* promoter hypermethylation functionally mirrors the defect seen with *MLH1* loss by compromising DNA repair. *MGMT* silencing also fosters genomic instability, often occurring concurrently within CIMP [70].

Moreover, this epigenetic strategy extends to cell cycle regulators, such as *RASSF1A* and the Wnt pathway gatekeeper *APC*. While *APC* is classically inactivated by somatic mutation in the traditional chromosomal instability (CIN) pathway, its silencing via promoter methylation in sporadic cases achieves the same functional endpoint: unchecked Wnt/β-catenin signalling. Thus, the tumour utilizes a singular epigenetic mechanism, promoter hypermethylation, to systematically dismantle diverse protective barriers, ranging from DNA repair (*MLH1*, *MGMT*) to cell cycle control (*RASSF1A*, *APC*), thereby driving the multifaceted progression of colorectal carcinogenesis [71,72].

### 4.2. The Mediterranean Diet: Epigenetic Restoration via Methyl Donors and Bioactive Compounds

The Mediterranean Diet (MD) is distinguished by a nutritional profile abundant in plant-based foods, healthy fats, and diverse bioactive compounds. Characterized by the high consumption of fruits, vegetables, legumes, whole grains, and olive oil, along with moderate intake of fish, this dietary pattern serves as a potent reservoir of essential micronutrients and phytochemicals [73]. Specifically, it ensures a robust supply of B-complex vitamins, including folate, riboflavin, pyridoxine, cobalamin, and essential trace elements such as selenium [61]. Beyond these micronutrients, the MD is defined by its rich content of polyphenols, including resveratrol, quercetin, and epigallocatechin-3-gallate (EGCG), as well as anti-inflammatory omega-3 fatty acids and dietary fiber [61].

Folate and vitamins B2, B6, and B12, abundant in green leafy vegetables, are crucial for the synthesis of S-adenosylmethionine (SAM), the universal substrate required by DNA methyltransferases (DNMTs) [61]. While adequate SAM availability is essential for maintaining global methylation homeostasis and genomic stability, it is important to distinguish this from aberrant promoter-specific hypermethylation. Dysregulation of one-carbon metabolism may simultaneously promote global hypomethylation while facilitating locus-specific hypermethylation at tumour suppressor gene promoters through altered DNMT targeting and chromatin context [74,75].

Polyphenols like resveratrol (grapes/wine) and curcumin (turmeric) act as natural inhibitors of DNA methyltransferases (DNMTs). Studies have shown that they can demethylate and reactivate silenced TSGs, such as *p16* and *RASSF1A* [47,76]. Furthermore, curcumin has been observed to act synergistically with folate and selenium to suppress DNMT expression, specifically aiding in the restoration of *MLH1* activity [47]. EGCG (epigallocatechin-3-gallate), the principal catechin in green tea—another polyphenol, functions as a potent epigenetic modulator by directly inhibiting DNA methyltransferases (DNMTs) [61]. It binds to the catalytic domain of enzymes such as DNMT3B, a process that facilitates the demethylation and transcriptional reactivation of silenced tumour suppressor genes, including *p16INK4a*, *MGMT*, and *RARB* [64]. Importantly, the mechanisms by which polyphenols modulate DNMT activity are compound-specific. EGCG has been shown to directly interact with DNMT catalytic domains, whereas curcumin and resveratrol primarily exert indirect effects by modulating DNMT expression, inflammatory signalling pathways, and redox status, resulting in context-dependent epigenetic reprogramming rather than direct enzymatic inhibition.

Fish oil, a rich source of omega-3 polyunsaturated fatty acids (PUFAs), exerts a notable influence on the epigenetic landscape of colorectal cancer cells by modulating the methylation status of key protein-coding genes. Current evidence suggests that omega-3 PUFAs primarily influence DNA methylation indirectly, through attenuation of inflammation, modulation of oxidative stress, regulation of DNMT expression, and effects on one-carbon metabolism, rather than acting as direct demethylating agents. Research indicates that treatment with these fatty acids can reverse the aberrant promoter hypermethylation of critical tumour suppressors, including *COX-2*, *CDKN2A* (*p16INK4a*) and *PTEN* [77]. By facilitating the demethylation and subsequent re-expression of these genes, omega-3 PUFAs restore essential regulatory pathways that govern cell cycle arrest, apoptosis, and anti-inflammatory signalling, effectively counteracting the gene silencing that drives tumorigenesis [77].

### 4.3. The Western Diet: Genomic Instability and Hypermethylation

In contrast, the Western diet, dominated by saturated fats, refined sugars, and processed meats, has been linked to hypomethylation of the *IGF2* differentially methylated region (DMR0). This epigenetic aberration is a surrogate marker for the Loss of Imprinting (LOI) of *IGF2*, a critical growth factor. Normally imprinted, *IGF2* is expressed only from the paternal allele; however, diet-induced hypomethylation can lead to biallelic expression (LOI), which drives pathological cell proliferation and is independently associated with higher mortality in colorectal cancer patients [62]. Chronic exposure to pro-inflammatory and insulin-resistance states induced by such diets amplifies oxidative stress, promoting aberrant methylation landscapes that parallel those seen in malignant transformation [41,78].

High-fat, frequent consumption (and thus a lifelong Western-style diet) has been shown to reduce global DNA methylation in colonic mucosa significantly. Experimental models demonstrate that a Western diet, characterized by high fat and low calcium intake, leads to a progressive loss of global methylation, mirroring the age-related epigenetic drift observed in CRC [79].

High alcohol consumption interferes with folate absorption and metabolism. This “double hit” (low folate and high alcohol) is strongly associated with promoter hypermethylation of genes like *APC* and *p16*, creating a field defect that predisposes to cancer [56].

Red meat intake promotes an inflammatory context that may indirectly alter methylation patterns. Studies suggest that high red meat consumption correlates with specific methylation signatures, potentially through oxidative stress mechanisms that recruit DNMTs to TSG promoters [80,81,82,83].

Low fiber and high protein content drastically restructure the gut microbiota, promoting a state of dysbiosis that generates toxic metabolites with potent epigenetic activity. A sharp reduction in fermentable carbohydrates leads to diminished production of beneficial short-chain fatty acids (SCFAs) like butyrate, a critical inhibitor of histone deacetylases (HDACs). This loss of butyrate-mediated HDAC inhibition contributes to a restrictive chromatin state and hinders the expression of TSGs. Concurrently, the elevated sulphur content (often from processed meats) fosters the overgrowth of sulphate-reducing bacteria. This results in the overproduction of hydrogen sulphide (H_2_S), a gas that acts as a genotoxic and inflammatory agent [84]. High luminal H_2_S has been experimentally shown to activate the NF-κB signalling cascade and to cause hypermethylation of the *APC* promoter by recruiting DNA methyltransferases (DNMT1 and DNMT3B), thus creating a direct molecular link between the Western diet, microbial metabolites, and the epigenetic silencing of key protective genes.

Diet and lifestyle also appear to modulate LINE-1 methylation: folate-rich and Mediterranean-type diets are linked to higher LINE-1 methylation levels, whereas Western dietary patterns and alcohol intake promote hypomethylation [85,86].

Many of these diet-associated epigenetic alterations are supported predominantly by associative epidemiological data or experimental models and are likely mediated through intermediary processes such as chronic inflammation, insulin resistance, oxidative stress, microbiota dysbiosis, and altered metabolite signalling, rather than direct dietary effects on DNA methylation machinery.

### 4.4. Systemic Impact: Population-Level Epigenetics and Dietary Interventions

Population-level data are consistent with these underlying mechanisms. Within the EPIC-Italy cohort, for instance, investigators observed that high adherence to the Mediterranean Diet correlated with differential methylation of inflammation-related genes, including *IL6*, *TNF*, and *CXCL12*, in circulating leukocyte DNA. Using a targeted epigenome-wide approach, CpG sites within these loci exhibited methylation differences exceeding 1% between colorectal cancer cases and controls, suggesting a functional link between diet, systemic inflammation, and epigenetic remodelling. Such findings illustrate, at a population level, how dietary adherence may attenuate carcinogenic risk by reprogramming methylation patterns in pro-inflammatory pathways [46].

These complex, nutrigenomic interactions thus underscore the multifactorial nature of epigenetic regulation, in which diet acts not merely as a modifier of metabolism but also as a transcriptional architect involved in genomic stability [43,46,47].

Following this logic, the interplay between dietary patterns and DNA methylation defines a crucial pillar in colorectal cancer prevention. Epigenetic events such as promoter methylation of *MLH1*, *APC*, *CDKN2A*, *MGMT*, and *RASSF1A* constitute molecular fingerprints of early carcinogenic processes, while dietary interventions, particularly the Mediterranean Diet, offer an avenue to counteract these aberrations. The capacity of polyphenols and methyl donors to modulate DNMT activity situates nutrition as a form of epigenetic therapy, capable of rewriting the molecular memory of cells exposed to carcinogenic stress [48,49]. The genes presented, along with their biological functions, methylation status in CRC, and corresponding dietary and epigenetic modulation mechanisms, are summarized in Table 1.

## 5. Micro-RNAs at the Crossroad Between Diet and CRC

microRNAs (miRNAs) are small non-coding RNAs that act as post-transcriptional regulators of gene expression, playing essential roles in intestinal homeostasis and contributing to broader aspects of an organism’s health, including immune and metabolic balance. In cancer, miRNAs are often classified into tumour suppressor miRNAs and oncogenic miRNAs depending on their expression levels and effects; however, this is only a simplified model, as the effect of miRNAs can depend on the total activity of their regulated genes [98]. In colorectal carcinogenesis, miRNAs participate at every stage of tumour development, from initiation and inflammation to metastasis, by targeting key oncogenic and tumour-suppressor pathways, including Wnt/β-catenin, PI3K/AKT, KRAS, and p53 [19]. Although miRNAs are often encoded by genes located in unstable regions, which leads to their deletion and consequently a lack of expression, in CRC, miRNAs are usually overexpressed due to an amplification of their encoding genes or because their promoters are active constitutively [99]. Nonetheless, tumour suppressor miRNAs are equally important since their decreased expression has been associated with an increased expression of several genes involved in tumour progression in CRC [98].

A growing number of miRNAs are being investigated in CRC, either in cohorts of patients, animal models or cell lines. Additionally, in silico studies constantly provide new potential targets, which causes substantial difficulty in selecting the most relevant miRNAs for the development of diagnostic, staging and prognostic biomarkers. With high-throughput methods now available, hundreds of miRNAs are being investigated in relation to CRC alone. In the following subchapters, we will focus on those miRNAs whose expression has been linked to diet regulation; however, extensive reviews of miRNA biogenesis, pathways and involvement in CRC are also available [100,101,102].

### 5.1. Vitamin D and the VDR Axis: The Odd One Out

Emerging evidence presents the intricate interplay between Vitamin D metabolism and miRNA regulation. Vitamin D exerts remarkable anti-inflammatory and anti-proliferative effects through the activation of the vitamin D receptor (VDR). Several miRs, such as miR-21, miR-22, and miR-627, have been identified as downstream targets of Vitamin D-VDR signalling [103,104]. Namely, miR-21, an oncomiR often upregulated in tumour cells to inhibit tumour suppressors like PTEN and PDCD4, is downregulated by Vitamin D supplementation [105]. Conversely, miR-22 and miR-627, which possess tumour-suppressive properties, are upregulated by calcitriol (the active form of Vitamin D), thereby mediating anti-proliferative effects in colon cancer cells [106].

Despite the established chemopreventive effects of Vitamin D and the protective nature of the Mediterranean diet, several observational studies reveal a significant so-called “Vitamin D paradox”—which describes a low serum 25(OH)D in the context of high sun exposure [44,107]. A large meta-analysis by Manios et al. (2018) [108], encompassing data from over 630,000 individuals, reported mean 25(OH)D serum concentrations below 50 nmol/L, indicating low Vitamin D status, across Southern Europe and the Eastern Mediterranean. This deficiency, particularly pronounced in females and adolescents, suggests that factors beyond sun exposure, such as limited dairy consumption or modern indoor lifestyles, prevent the full utilization of Vitamin D’s protective epigenetic potential [108]. Furthermore, Trovato et al., in a study published during the same year, utilizing a rat model, demonstrated that the high-fat components often co-occurring with modern Western diets can impair muscle metabolism and reduce the trophic action of Vitamin D on muscle fibers [105]. Collectively, these findings emphasize that the protective benefits of the Mediterranean diet against CRC are complex and may be undermined by concurrent sub-optimal Vitamin D status, while also underlining the creeping influence of pro-inflammatory components of the Western diet.

### 5.2. The Mediterranean Diet: Restoring Tumour Suppression by Way of Polyphenols

It is important to distinguish whether diet-associated miRNA alterations arise from direct epigenetic regulation (e.g., promoter methylation or chromatin remodelling) or represent secondary responses to modulation of inflammatory, metabolic, or oncogenic signalling pathways. In many cases, observed miRNA changes reflect indirect downstream effects rather than primary epigenetic targeting.

The Mediterranean Diet (MD), as presented earlier, is characterized by high intakes of antioxidant-rich foods (fruits, vegetables, extra virgin olive oil, nuts) and thus is abundant in bioactive compounds, such as polyphenols, which act as potent modulators with anti-cancer effects. Specific components standard in the MD, such as resveratrol (grapes) and curcumin (turmeric), actively upregulate tumour-suppressive miRNAs that are often silenced in CRC [109,110].

Resveratrol (found in grapes and wine) is a polyphenol that exerts dual protective mechanisms. It downregulates oncomiRs such as miR-21 [111] and the miR-17-92 cluster [111,112,113]. Simultaneously, it upregulates tumour-suppressor miRNAs, including miR-663 (which targets TGFβ transcripts to reduce metastatic potential) and miR-34a (a key p53-effector that induces apoptosis) [114,115].

Curcumin shares the anti-inflammatory profile of the MD, despite being a spice. It has been shown to downregulate the oncomiR miR-21 via the AP-1 transcription factor, thereby restoring PDCD4 expression [116]. Furthermore, it induces the downregulation of the oncogenic miR-27a, which suppresses specificity proteins essential for angiogenesis [117].

Quercetin (found in onions and apples) is a flavonoid often tied to MD and has been shown to upregulate miR-146a, which facilitates the downregulation of the NF-κB inflammatory pathway, counteracting the pro-inflammatory state often induced by Western dietary habits [118].

Omega-3 PUFAs such as docosahexaenoic acid (DHA) can upregulate miR-126 through promoter demethylation in CRC cell lines, showing an antiangiogenic effect [119,120]. Additionally, downregulation of miR-27b, miR-93, miR-497, as well as miR-18a and miR-19b (part of the miR-17-92a oncogenic cluster) has been observed. A combination of fish oil PUFAs and a high fiber diet also downregulates oncogenic miRNAs, namely miR-16, miR-21, miR-26b and miR-27b [119].

A high fiber diet leading to high doses of butyrate has been associated with a decrease in oncogenic miRNAs belonging to the miR-106b family and the miR-17-92a cluster in CRC cell lines. Butyrate can also induce tumour suppressor miR-139 and miR-542 in cell lines, which reduce proliferation and induce apoptosis [119].

### 5.3. The Western Diet: Carbohydrates, Red Meat, and Oncogenic Signalling

In contrast to the MD, the Western dietary pattern is characterized by a high intake of refined carbohydrates and red/processed meats, which appears to drive an oncogenic miRNA profile characterized by the silencing of tumour suppressors.

Red and processed meat, especially their excessive intake, is a hallmark of the Western diet and has been linked to specific epigenetic alterations. A high red meat diet has been shown to upregulate the miR-17-92 cluster and miR-21 in rectal mucosa, both of which are oncomiRs known to be upregulated in CRC [119,121,122].

High-fat intake, another staple of the Western lifestyle, has been observed to downregulate tumour suppressors miR-143 and miR-145 via EGFR signalling. The loss of these miRNAs leads to the upregulation of their target oncogenes, KRAS and MYC, promoting tumorigenesis [123]. Deoxycholic acid (DHA), a gut microbiome metabolite induced by a high-fat diet, can decrease miR-199a-5p levels, leading to an overexpression of its target, CAC1, which in turn contributes to tumorigenesis. Experimental evidence showed that overexpressed miR-199a-5p inhibits CRC cell proliferation and reverses drug resistance, providing a potential therapeutic target [124].

Omega-6 PUFAs as part of a Western diet tend to have the opposite effect of omega-3 PUFAs in the Mediterranean diet, when consumed excessively. Although PUFAs have been linked for years with a tumoricidal action, conflicting data is still coming to light, which could be attributed to the distinct chemical compounds being investigated, their different metabolization, the various models and methods used. A main subject of controversy has been linoleic acid (LA), which was associated both with increased and reduced frequency of CRC in different studies [125,126,127]. Interestingly, a case–control study found a protective role for LA in CRC, alongside oleic acid and α-linoleic acid, which are usually part of a Mediterranean diet [126]. Another study pinpointed LA as a cause of miR-494 upregulation, which led to the suppression of MYCC and PGC1α and produced cancer dormancy in the CT26 CRC mouse cell line. In human tissue, miR-494 was highly expressed in cases with delayed liver metastases, but showed low expression in cases with current liver metastases or no metastases [128]. More studies are needed to elucidate the relationship between certain PUFAs, CRC and the microRNAs involved.

High carbohydrate and sucrose intake was associated with distinct miRNA signatures in an extensive population-based study of 1447 CRC cases. Using a miRNA microarray, the authors identified 250 miRNAs differentially expressed between CRC and normal colonic tissue for carbohydrate intake, 198 for sucrose intake, and 166 for both [129]. Notably, miR-1224-5p and miR-10b-3p showed significant differential expression associated with high sucrose consumption, suggesting that the glucose-metabolic characteristics of Western diets directly influence epigenetic regulation [130,131]. When looking at previously studied miRNAs, the authors found different associations than previously seen in the literature, which raises concern for the translational value of studies of this nature done on mice and cell lines [129]. Representative miRNAs studied in colorectal carcinogenesis are described in Table 2.

All these findings suggest that many Western diet-associated miRNA alterations represent secondary epigenetic responses to metabolic and inflammatory perturbations rather than primary oncogenic drivers, underscoring the need for cautious interpretation when translating experimental miRNA data into clinical biomarkers.

## 6. DNA Methylation-Driven Regulation of microRNA Genes

While DNA methylation and microRNA expression are often studied as distinct phenomena, they are functionally intertwined. A considerable number of miRNA genes reside within CpG island-rich regions, making them susceptible to the same methylation pressures that affect protein-coding genes, as presented in the previous chapters. This creates a “double-hit” scenario: epigenetic modulation of a miRNA gene leads to unchecked translation of its oncogenic mRNA targets, thereby linking environmental and dietary exposures to gene silencing in CRC [140].

When promoter CpG islands become hypermethylated, transcription of tumour-suppressive miRNAs is repressed [141]. This phenomenon is particularly prominent in CIMP-high colorectal cancers, where widespread promoter methylation affects both protein-coding and non-coding loci [142].

Recent genome-wide analyses identified several miRNAs recurrently silenced by DNA methylation in CRC: miR-34b/c—a direct *p53*-regulated miRNA cluster; its methylation blocks apoptosis and cell-cycle arrest [143]; miR-137—hypermethylated in CIMP-positive and BRAF-mutated CRCs, while reactivation suppresses Wnt/β-catenin signalling through RNF4 inhibition [144]; miR-143/145 cluster—frequently methylated in both adenomas and carcinomas, acts on KRAS and IGF1R pathways [102,145,146] and miR-342, miR-124, and miR-129—methylation targets linked to serrated pathway lesions and inflammation-associated carcinogenesis [100,147].

This coordinated repression of tumour-suppressive miRNAs amplifies oncogenic signalling and reinforces the CIMP phenotype characteristic of proximal and serrated colorectal tumours [148].

### 6.1. Diet as a Modulator of miRNA Methylation

Dietary patterns influence the methylation potential and epigenetic machinery through both nutrient-dependent and inflammation-mediated mechanisms.

One-carbon metabolism—nutrients such as folate, methionine, choline, and vitamin B12 determine the availability of S-adenosylmethionine (SAM), the universal methyl donor.

Deficiency induces global hypomethylation but paradoxically promotes locus-specific hypermethylation, including at miRNA promoters (miR-34b/c, miR-137) [149].

Adequate dietary methyl donors maintain regular miRNA expression, whereas an imbalance can accelerate tumour-associated silencing [150].

#### 6.1.1. The Western Diet: Driving the Methylation of Tumour-Suppressor miRNAs

Diets rich in saturated fat, red and processed meat, and refined carbohydrates promote low-grade inflammation and DNMT upregulation [151]. This increases promoter hypermethylation of tumour-suppressive miRNAs and enhances expression of inflammatory oncomiRs (miR-21, miR-155, miR-135b) [152]. These diet-induced miRNA changes converge on PI3K/AKT and NF-κB pathways, fostering proliferation, invasion, and resistance to apoptosis [119].

Thus, under Western dietary conditions (characterized by low levels of methyl donors and high inflammation), promoters of tumour suppressors, such as miR-34b/c and miR-137, become hypermethylated. This silencing blocks their ability to induce apoptosis and to suppress Wnt/β-catenin signalling, a hallmark of the CIMP-high pathway, often observed in proximal tumours [32,119].

#### 6.1.2. The Mediterranean Diet: Reactivation via Demethylation

Conversely, as stated in previous chapters, the MD offers a mechanism for “epigenetic rescue.” Bioactive compounds such as polyphenols and microbial SCFAs not only upregulate miRNA expression but also physically modify the chromatin landscape of miRNA genes.

Mediterranean and plant-based diets, rich in polyphenols (such as resveratrol, quercetin and EGCG), omega-3 fatty acids, and fermentable fibres that produce short-chain fatty acids (SCFAs) such as butyrate, can inhibit DNMT1 and HDACs, facilitating demethylation and reactivation of silenced miRNAs [47]. For instance, butyrate restores miR-143 and miR-34a expression, while resveratrol and EGCG counteract miR-21 overexpression, shifting the balance toward anti-tumorigenic signalling [153].

### 6.2. The Diet–miRNA Methylation–CIMP Axis

The coexistence of miRNA promoter hypermethylation and global CpG island methylation suggests that CIMP and miRNA silencing share a common epigenetic origin [154]. Dietary and metabolic conditions that favour DNMT activation (high-fat, low-folate) can intensify both CIMP and miRNA methylation, reinforcing the epigenetic blockade of differentiation and apoptosis. On the other hand, exposure to “epigenetically protective diets” (Mediterranean, high-fiber, polyphenol-rich) may attenuate this phenotype by downregulating DNMT/HDAC and increasing TET-mediated DNA demethylation [47].

Recently emerging research also links these diet-driven miRNA methylation signatures with circulating biomarkers detectable in liquid biopsy [154,155,156,157]. Tumours exhibiting hypermethylation of miR-34b/c or miR-137 frequently release corresponding methylated cfDNA fragments and dysregulated circulating miRNAs (miR-21, miR-92a, miR-135b), suggesting a shared epigenetic imprint between diet, tumour biology, and systemic circulation [158]. This diet-based epigenetic remodelling is summarized in Table 3, which highlights the methylation status of key miRNA genes.

## 7. Liquid Biopsy-Based Epigenetic Biomarkers in CRC: DNA Methylation, miRNAs and miRNA Gene Methylation

Epigenetic modifications represent one of the most promising sources of biomarkers for liquid biopsy-based detection and risk stratification in CRC. Diet-dependent changes in DNA methylation, circulating miRNAs, and methylation of miRNA-coding genes can be captured in cell-free DNA (cfDNA) or extracellular vesicles, providing a minimally invasive window into tumour biology and environmental modulation. These molecular signatures may support both early diagnosis and prevention-oriented monitoring, especially in individuals exposed to specific dietary patterns.

Methodologically, circulating miRNAs are quantified using approaches such as quantitative PCR (qPCR), digital droplet PCR (ddPCR), next-generation sequencing (NGS), and analysis of exosomal versus free-circulating fractions, each with distinct analytical sensitivities and translational readiness. Similarly, cfDNA methylation assessment relies on methylation-specific PCR–based assays or sequencing-based methods, with only a subset currently validated for clinical application.

### 7.1. DNA Methylation Biomarkers Detectable in Liquid Biopsy

Aberrant DNA methylation is a hallmark of CRC progression and can be measured in cfDNA with high analytical sensitivity. Established markers such as SEPT9 already serve as clinical screening tools [170], while additional genes known to be methylated in tumour tissue—*MLH1*, *APC*, *CDKN2A/p16*, *MGMT*, *RASSF1A*, *SFRP1/2*, and global LINE-1 hypomethylation—are increasingly reported in plasma-derived cfDNA [170,171].

Dietary exposures influence many of these methylation changes through modifications of one-carbon metabolism, inflammation, and microbiota-derived metabolites. Consequently, cfDNA methylation profiles may function as diet-responsive biomarkers that reflect long-term environmental risk as well as tumour-derived alterations.

### 7.2. Circulating microRNAs as Non-Invasive Biomarkers

Circulating miRNAs are stable in blood and exhibit reproducible changes during colorectal tumorigenesis [172]. As highlighted earlier, several miRNAs commonly dysregulated in CRC tissue, such as miR-21, miR-17-92 cluster, miR-27a, miR-92a, miR-135b, miR-34a, miR-143/145, and miR-137, have been consistently detected in plasma or serum. Gasparello et al. proposed a nine-miRNA signature for the early diagnosis of CRC, which consisted of five up-regulated miRNAs (mir-1247-5p, mir-584-5p, mir-10a-5p, miR-483-5p, miR-425-3p), four down-regulated miRNAs (mir-15b-5p, mir-486-5p, mir-144-5p, mir-144-3p) and showed great specificity in their cohort [173]. A recent meta-analysis investigated the potential use of 90 microRNAs, focusing on 7 of the most studied ones, namely miR-23, miR-92, miR-21, miR-17, miR-150, miR-29 and miR-20. Of these, the first three showed the highest accuracy and sensitivity [174]. Notably, miR-21 and miR-92 were also part of a 6-miRNA signature proposed for diagnostic use over 10 years ago, but so far, no miRNA-based diagnostic test has been validated [175,176,177].

Importantly, many of these miRNAs are sensitive to dietary patterns (Table 3). Western-style diets enriched in saturated fats and red meat are associated with increased circulating miR-21, miR-27a, and miR-135b, whereas Mediterranean or plant-based diets tend to elevate tumour-suppressive miRNAs such as miR-34a, miR-143/145, miR-126, and miR-22. Because circulating miRNAs integrate both environmental exposures and tumour biology, they could represent a powerful tool for both risk assessment and disease monitoring.

### 7.3. Methylation of miRNA Genes in cfDNA

Promoter hypermethylation of tumour-suppressive miRNAs—such as miR-34b/c, miR-124, miR-137, miR-143/145, and miR-342 is a recurrent feature of CRC. Although many studies have traditionally focused on tissue samples, the review by Longo et al. (2025) highlights that these methylation marks can also be reliably detected in cfDNA, offering a dual-layer biomarker: loss of miRNA expression combined with a stable methylation signal in the circulation [100].

**Figure 3:**
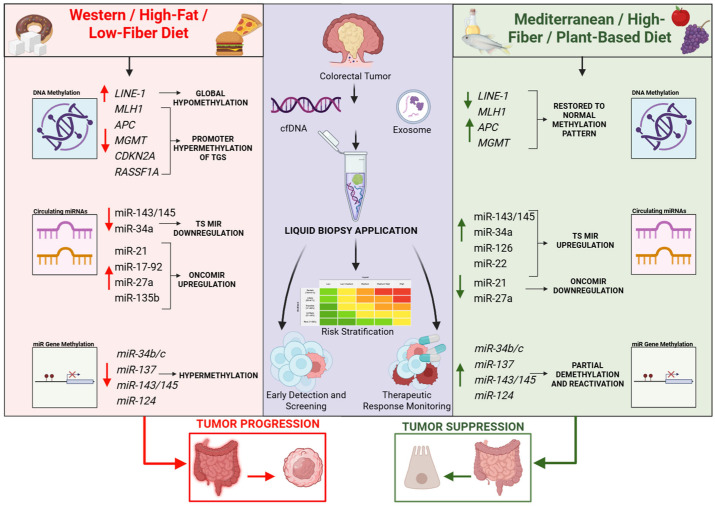
Diet-linked epigenetic signatures with potential clinical utility; These diet-linked epigenetic patterns, measurable through liquid biopsy, could support personalized risk prediction and the monitoring of preventive dietary interventions. Western/High-Fat Diet (**Left**): Characterised by global genomic instability (hypomethylation of LINE-1) and promoter hypermethylation of tumour suppressors (*MLH1*, *APC*, *MGMT*) and silencing of protective microRNAs (miR-34b/c, miR-137). Mediterranean/High-Fiber Diet (**Right**): Utilises bioactive compounds to inhibit DNA methyltransferases, restoring normal methylation patterns and reactivating silenced genes. Liquid Biopsy Application (**Centre**): These signatures can be captured non-invasively via cfDNA and circulating microRNAs to support early detection, risk stratification, and therapeutic monitoring of dietary interventions. Arrows indicate: ↑—upregulated expression; ↓—downregulated expression. Created in BioRender. Chindea, T. (2026) https://BioRender.com/28wycew.

Dietary factors that modulate DNMT activity (e.g., low folate, high-fat intake) or enhance demethylation pathways (e.g., SCFAs such as butyrate from high-fiber diets, polyphenols, or omega-3 fatty acids) may directly influence the methylation status of miRNA promoters, making this class of biomarkers particularly relevant for prevention-oriented liquid biopsy strategies. Diet-linked epigenetic signatures with potential clinical utility are summarized in Figure 3.

## 8. Future Perspective

Given the projected rise in colorectal cancer incidence, future studies will increasingly focus on comprehensive epigenetic profiling of this disease. Understanding how dietary compounds can reverse epigenetic marks is thus crucial. This knowledge will guide the development of “nutri-epigenetic” strategies targeting specific alterations, offering a promising avenue to overcome resistance to conventional treatments.

In this context, the field is rapidly transitioning from descriptive epigenetics toward functional and predictive epigenomics, aiming to identify causal epigenetic drivers rather than passive bystander marks. Advanced technologies such as single-cell multi-omics, spatial epigenomics, and long-read methylome sequencing are expected to uncover epigenetic dependencies unique to colorectal cancer subtypes, thereby enabling stratified and mechanism-driven nutritional interventions.

Moreover, the emergence of targeted epigenetic interventions, including the combination of dietary bioactive compounds (e.g., resveratrol, curcumin) with clinical DNMT inhibitors, births a new era of combinatorial therapy. These approaches hold the potential not only to sensitize tumour cells to chemotherapy but also to open new ways for chemoprevention strategies that target multiple tumorigenic pathways simultaneously (such as the Wnt/β-catenin and inflammatory pathways), thereby enhancing efficacy and preventing recurrence.

Notably, such combinatorial strategies align with the concept of epigenetic priming, whereby dietary compounds recondition the chromatin landscape, rendering malignant cells more vulnerable to subsequent pharmacological or immune-mediated attack. This approach reframes nutrition as an active epigenetic co-therapeutic force, capable of reshaping transcriptional plasticity, attenuating adaptive resistance, and constraining tumour evolutionary trajectories.

The advancement of liquid biopsy technologies and integrative omics analyses marks a significant step forward in fighting early-onset and sporadic CRC. By analysing circulating tumour DNA (ctDNA) methylation patterns (e.g., *SEPT9*, *MLH1*) and circulating microRNAs (e.g., miR-21, miR-92a), liquid biopsies provide a non-invasive way to detect CRC early, monitor its progress, and determine prognosis. Liquid biopsy-based epigenetic profiling enables longitudinal surveillance of tumour epigenomic plasticity, capturing therapy-induced shifts and early molecular signs of relapse well before clinical manifestation. This real-time monitoring defines epigenetic biomarkers as dynamic indicators of tumour evolution, rather than static diagnostic endpoints.

Additionally, a promising new area is the detection of methylated microRNA genes within circulating cell-free DNA. Emerging research shows that the promoter hypermethylation of tumour-suppressive miRNAs, such as miR-34b/c and miR-137, can be detected systemically, mirroring the epigenetic silencing in the primary tumour. These methylated miR biomarkers serve as a powerful dual epigenetic signature, indicating both the DNA methylation process and the loss of post-transcriptional regulation, potentially offering greater sensitivity and specificity than single markers.

Furthermore, integrating genomic, transcriptomic, and epigenomic data with metagenomics (microbiomics) promises to provide a holistic understanding of the “hologenome” in CRC biology. This approach is essential, as the gut microbiome acts as the key mediator between diet and the host epigenome. By shaping host chromatin architecture through microbially derived metabolites, the gut microbiota emerges as a central epigenetic regulator, capable of modulating cancer susceptibility, immune tone, and therapeutic responsiveness. Integrating microbiome-informed epigenetic signatures into predictive models may ultimately enable precision prevention frameworks, in which dietary modulation is tailored to individual epigenomic and microbial configurations. Deciphering this crosstalk opens new avenues for the discovery of novel biomarkers, thereby enhancing the precision of CRC prevention.

Altogether, these innovative strategies underscore the importance of a multifaceted approach to improving diagnosis, treatment, and prognosis for CRC patients, highlighting a future in which personalized precision nutrition becomes a reality. Moreover, the convergence of nutriepigenetics, liquid biopsy-driven epigenomic surveillance, and hologenomic integration heralds a transformative shift toward anticipatory, systems-level oncology, where colorectal cancer is intercepted, reprogrammed, and prevented through informed manipulation of the epigenetic landscape.

## 9. Conclusions

Colorectal cancer represents a paradigm of gene–environment interaction in which diet plays a decisive role in shaping the epigenome. The evidence reviewed here demonstrates that DNA methylation, microRNA expression, and especially the methylation of microRNA-coding genes represent central regulatory layers influenced by Western and Mediterranean dietary patterns. These diet-sensitive epigenetic alterations not only contribute to tumour initiation and progression but also leave measurable traces in circulation, detectable through modern liquid biopsy approaches.

Together, these findings support the development of diet-responsive epigenetic biomarkers that could improve risk stratification, early detection, and monitoring. Moreover, harnessing these biomarkers may transform CRC management into a truly personalized and nutria-epigenetically informed domain.

Future research should integrate nutritional interventions with epigenetic profiling to advance precision prevention strategies in colorectal cancer. As diet-modulated epigenetic markers continue to be validated, a future in which colorectal cancer screening and intervention are tailored to an individual’s metabolic and epigenetic profile becomes increasingly plausible, marking an important step toward next-generation, biomarker-driven screening programs.

## Figures and Tables

**Table 1:** Representative genes frequently affected by DNA methylation in colorectal carcinogenesis and their dietary/biological modulation.

Gene	Biological Function	Role of Methylation in CRC	Possible Dietary/Epigenetic Modulation	References
*MLH1*	DNA mismatch-repair enzyme maintaining replication fidelity.	Gene silencing leading to microsatellite instability (MSI) phenotype; common in sporadic proximal CRCs.	Adequate folate and vitamin B12 maintain one-carbon metabolism and prevent hypermethylation; Curcumin and resveratrol may demethylate *MLH1* promoter in vitro.	[52,54,64].
*APC*	Regulates β-catenin degradation in the Wnt pathway.	Leads to Wnt pathway activation and uncontrolled proliferation.	Folate deficiency enhances *APC* methylation; Polyphenols may reactivate *APC* transcription.	[55,72,87,88].
*CDKN2A (p16INK4a)*	Cyclin-dependent kinase inhibitor controlling the G1 to S transition.	Suppresses cell-cycle checkpoint leading to unrestrained proliferation.	Resveratrol and sulforaphane inhibit DNMTs and restore p16 expression in colon cancer cells.	[57,89,90].
*MGMT*	DNA repair enzyme removing O6-methylguanine lesions.	Results in reduced DNA repair capacity and increased mutation burden.	Folate supplementation may maintain methylation balance; green-tea compounds demethylate promoter in vitro.	[58,71,91,92].
*RASSF1A*	Regulates apoptosis and microtubule stability.	Silences apoptosis-inducing signalling causing enhanced tumour cell survival.	Resveratrol and curcumin shown to demethylate the promoter and restore expression.	[76,93].
*SFRP1/SFRP2*	Secreted antagonists of Wnt signalling.	Results in constitutive Wnt pathway activation.	Polyphenols attenuate Wnt signalling and may demethylate the promoters.	[59,94].
*CXCL12 (SDF-1)*	Chemokine controlling leukocyte trafficking and angiogenesis.	Suppresses immune surveillance, promotes tumour invasion.	High adherence to MD associated with altered methylation of *CXCL12* in EPIC-Italy cohort.	[46,95].
LINE-1 (repetitive element)	Surrogate marker of global DNA methylation.	Global hypomethylation correlates with genomic instability and poor prognosis.	Folate-rich diets and polyphenol intake maintain LINE-1 methylation levels.	[60,96,97].

**Table 2:** Representative miRNAs studied in colorectal carcinogenesis and their dietary modulation.

MicroRNA	Expression in CRC and Type	Biological Function	Dietary Modulation (Western vs. Mediterranean Diet)	References
miR-21	↑ (oncomiR)	Promotes proliferation and invasion.	Upregulated by high-fat and red meat consumption. Downregulated by vitamin D, curcumin, and resveratrol.	[111,119]
miR-17-92 cluster	↑ (oncomiR)	Promotes cell cycle progression.	Upregulated by red meat consumption and obesity. Downregulated by resveratrol and curcumin.	[113,132,133]
miR-34a	↓ (tumor suppressor)	Induces apoptosis and senescence.	Upregulated by resveratrol, quercetin, and curcumin analogues. Downregulated by calorie restriction	[134]
miR-27a	↑ (oncomiR)	Plays a role in proliferation, apoptosis, invasion, angiogenesis.	Upregulated by high-fat diet and obesity. Downregulated by curcumin and quercetin.	[135,136]
miR-627	↓ (tumor suppressor)	Targets a histone demethylase.	Upregulated by vitamin D (calcitriol). Downregulated by vitamin D deficiency.	[104]
miR-126	↓ (tumor suppressor)	Inhibits tumor growth and metastasis.	Upregulated by polyphenols and fish oils. Downregulated by obesity.	[120,137,138,139]

Arrows indicate: ↑—upregulated expression; ↓—downregulated expression.

**Table 3:** Diet-sensitive miRNAs and their methylation status in colorectal cancer.

miRNA	Functional Role	Target/Pathway	Methylation/Epigenetic Mechanism	Key References
miR-34b/c	Tumor suppressor	*p53*, apoptosis, cell cycle	Promoter CpG island hypermethylation (CIMP-high); reactivated by butyrate, polyphenols	[32,143,159].
miR-137	Tumor suppressor	*Wnt/β-catenin*, *RNF4*	Hypermethylated in BRAF-mutant/CIMP+ CRC; reversible by folate/B12 repletion	[143,160].
miR-143/145	Tumor suppressor cluster	*KRAS*, *IGF1R*, *ERK*	DNMT1-mediated promoter methylation; demethylated by SCFAs (butyrate)	[161].
miR-342	Tumor suppressor	*DNMT1 feedback loop*	Aberrant methylation in serrated lesions; diet-sensitive	[146,147,162].
miR-124/miR-129	Tumor suppressor	*STAT3*, *PI3K/AKT*	Methylated in CIMP-high and inflammatory CRC	[147,163,164].
miR-21	OncomiR	*PTEN*, *NF-κB*, *PI3K/AKT*	Upregulated under Western diet; induced by inflammation rather than methylation	[165,166,167].
miR-135b	OncomiR	*APC/Wnt*	Induced by high-fat diet, inflammatory cytokines; not silenced by methylation	[121].
miR-200c	Tumor suppressor	*EMT regulators (ZEB1/2)*	Promoter methylation reversible by polyphenols (resveratrol, EGCG)	[168,169].

## Data Availability

No new data were created or analyzed in this study. Data sharing is not applicable to this article.

## Abbreviations

The following abbreviations are used in this manuscript:

5-mC	5-Methylcytosine
AGE	Advanced Glycation End products
APC	Adenomatous Polyposis Coli
AKT	Protein Kinase B
BCL-2	B-cell Lymphoma 2
BMI	Body Mass Index
BRAF	v-Raf Murine Sarcoma Viral Oncogene Homolog B
CDKN2A	Cyclin-Dependent Kinase Inhibitor 2A (p16)
cfDNA	Cell-Free DNA
CIMP	CpG Island Methylator Phenotype
CIN	Chromosomal Instability
CMS	Consensus Molecular Subtypes
COX-2	Cyclooxygenase-2
CRC	Colorectal Cancer
ctDNA	Circulating tumour DNA
CXCL12	C-X-C Motif Chemokine Ligand 12
DHA	Docosahexaenoic Acid
DNA	Deoxyribonucleic Acid
DNMT	DNA Methyltransferase
EGCG	Epigallocatechin-3-Gallate
EGFR	Epidermal Growth Factor Receptor
EMT	Epithelial-Mesenchymal Transition
eoCRC	Early-Onset Colorectal Cancer
EPA	Eicosapentaenoic Acid
EPCAM	Epithelial Cell Adhesion Molecule
ESR1	Estrogen Receptor 1
EVs	Extracellular Vesicles
FADS	Fatty Acid Desaturase
FAP	Familial Adenomatous Polyposis
GSTP1	Glutathione S-Transferase Pi 1
H2S	Hydrogen Sulfide
HDAC	Histone Deacetylase
HNPCC	Hereditary Non-Polyposis Colorectal Cancer (Lynch Syndrome)
IBD	Inflammatory Bowel Disease
IGF	Insulin-Like Growth Factor
IGF-1	Insulin-Like Growth Factor 1
IL-6	Interleukin-6
KRAS	Kirsten Rat Sarcoma Viral Oncogene Homolog
LINE-1	Long Interspersed Nuclear Element-1
LA	Linoleic Acid
LS	Lynch Syndrome
MBD	Methyl-CpG-Binding Domain Protein
MD	Mediterranean Diet
MGMT	O-6-Methylguanine-DNA Methyltransferase
miRNA/miR	microRNA
MLH1	MutL Homolog 1
MMR	DNA Mismatch Repair
MSH2/6	MutS Homolog 2/6
MSI	Microsatellite Instability
MSI-H	Microsatellite Instability-High
MTHFR	Methylenetetrahydrofolate Reductase
MYC	Myelocytomatosis Oncogene
NF-kB	Nuclear Factor Kappa B
NRF2	Nuclear Factor Erythroid 2-Related Factor 2
PDCD4	Programmed Cell Death 4
PGE2	Prostaglandin E2
PI3K	Phosphoinositide 3-Kinase
PMS2	PMS1 Homolog 2
